# S06-3: We Walk. Developing and pilot testing a dyadic intervention to promote outdoor walking in people after stroke: benefits, challenges and solutions

**DOI:** 10.1093/eurpub/ckae114.224

**Published:** 2024-09-26

**Authors:** Jacqui Morris, Linda Irvine, Tricia Tooman, Stephan Dombrowski, Brendan Mc Cormarck, Frederik van Wijk, Maggie Lawrence

**Affiliations:** University of Dundee; University of Dundee; University of Dundee; University of New Brunswick; University of Sydney; Glasgow Caledonian University; Glasgow Caledonian University

## Abstract

**Purpose:**

After stroke, physical activity (PA) levels are low, risking secondary stroke, cardiovascular disease, falls and poor mobility. Regular PA reduces these risks. Walking is a preferred PA option for stroke survivors, and social support influences uptake and maintenance of behaviours. We therefore developed WeWalk, a person-centred, dyadic peer-support behavioural intervention supported by walking buddies, to promote regular walking after stroke. This study evaluated participants’ experiences of WeWalk to refine it ready for effectiveness testing and implementation.

**Methods:**

Intervention: WeWalk involved facilitated face-to-face and telephone sessions with a researcher with behaviour change training, supported by intervention handbooks and diaries. Dyads agreed walking goals and plans, monitored progress, and developed strategies for maintaining walking.

**Evaluation:**

Data were collected through semi-structured interviews and facilitator fieldnotes during intervention delivery, and were analysed using thematic analysis, guided by a theoretical framework of acceptability.

**Results:**

We recruited 21 dyads comprising community-dwelling PWS and their walking buddies. Eighteen dyads completed exit interviews, one dyad was lost to follow-up and two withdrew with ill-health. We identified three themes: acceptability evolves with experience, mutuality, and person-centredness with personally relevant tailoring. As dyads recognised how WeWalk components supported walking, perceptions of acceptability grew. Effort receded as goals and enjoyment of walking together were realised. The dyadic structure provided accountability, and participants’ confidence developed as they experienced physical and psychological benefits of walking. WeWalk required careful facilitation. It worked best when dyads exhibited relational connectivity and mutuality in setting and achieving goals however in a few dyads agreeing and enacting mutual goals was more challenging. Tailoring intervention components to individual circumstances and values supported dyads in achieving meaningful goals.

**Conclusions:**

WeWalk is feasible and acceptable, illustrating the potential of dyadic interventions after stroke. However, its complexity requires considered implementation strategies. Data highlight the need for careful matching of dyads, facilitation of dyadic working by practitioners who can support development of dyadic relationships; and community structures linked to healthcare pathways that support implementation. We have worked with charities health services organisations to plan and develop these implementation strategies and report the challenges and potential solutions to delivering this complex intervention.

